# Cumulative Sport-Related Injuries and Longer Term Impact in Retired Male Elite- and Amateur-Level Rugby Code Athletes and Non-contact Athletes: A Retrospective Study

**DOI:** 10.1007/s40279-020-01310-y

**Published:** 2020-07-16

**Authors:** Karen Hind, Natalie Konerth, Ian Entwistle, Alice Theadom, Gwyn Lewis, Doug King, Paul Chazot, Patria Hume

**Affiliations:** 1 Department of Sport and Exercise Sciences, Durham University, 42 Old Elvet, Durham, DH1 3HN UK; 2 Wolfson Research Institute for Health and Wellbeing, Durham University, Queen’s Campus, Stockton, TS17 6BH UK; 3 National Institute for Stroke and Applied Neuroscience, Auckland University of Technology, Auckland, New Zealand; 4 Health and Rehabilitation Research Institute, Faculty of Health and Environmental Science, Auckland University of Technology, Auckland, New Zealand; 5 Sports Performance Research Institute New Zealand (SPRINZ), Faculty of Health and Environmental Science, Auckland University of Technology, Auckland, New Zealand; 6 Department of Biosciences, Durham University, Stockton Road, Durham, DH1 3LE UK

## Abstract

**Background:**

Rugby union and rugby league are popular team contact sports, but they bring a high risk of injury. Although previous studies have reported injury occurrence across one or several seasons, none have explored the total number of injuries sustained across an entire career. As the first to do so, the aim of this study was to report on cumulative injuries and their perceived long-term impact in retired rugby code athletes compared to athletes from non-contact sports.

**Methods:**

One hundred and eighty-nine former rugby code athletes (rugby union *n* = 145; rugby league *n* = 44) and 65 former non-contact athletes were recruited to the UK Rugby Health Project between September 2016 and December 2018. Details on sports participation, sports injuries and concussion history, sports injury-related surgeries, and previous and current health were obtained from a validated, online self-report questionnaire.

**Results:**

Former elite rugby code athletes (*n* = 83) reported more total injuries per player (median 39, IQR 35) than former amateur rugby code athletes (*n* = 106; median 23, IQR 30; *p* = 0.014) and non-contact sports athletes (*n* = 65; median 7.5, IQR 15; *p* < 0.001). Concussion was the most frequently reported injury for the elite and amateur rugby code groups, followed by upper/lower back and knee ligament injuries. These injuries also presented with the highest recurrence. Rugby code groups reported a higher continued impact of previous concussion, neck injuries, shoulder dislocation, ACL tears, and knee ligament injuries (*p* = 0.003–0.045). The reported prevalence of osteoarthritis was more than twofold greater in the elite rugby code group than in non-contact athletes (51% v 22%, *p* < 0.001). The prevalence of back pain and/or severe and regular joint pain was high across all groups (47–80%), particularly the elite rugby code group. The total number of joint injuries and sport injury-related surgeries was higher in those who reported current osteoarthritis and current severe and regular joint pain (*p* < 0.001–*p* = 0.028).

**Conclusion:**

Across multiple injury types, past participation in rugby union and rugby league, particularly at elite level, is associated with a high cumulative injury load and a continued impact of previous injuries post-retirement. Given the high number of reported concussions (and their recurrence) and associations between previous injuries during a player’s career and current musculoskeletal conditions, efforts should be prioritized to reduce the occurrence and recurrence of injuries in rugby codes at all levels of the sport. Strategies should also be developed for supporting the specific physical health needs of rugby code athletes post-retirement.

**Electronic supplementary material:**

The online version of this article (10.1007/s40279-020-01310-y) contains supplementary material, which is available to authorized users.

## Key Points

**Table:** 

Compared to age-matched, non-contact controls, and across multiple injury types, retired rugby code athletes, particularly at elite level, were 1.7–7.3 times more likely to report a given injury and 2.4–9.7 times more likely to report continued impact from a given injury.
Most former rugby code athletes (elite: 81%, amateur: 76%) reported at least one concussion, and concussion injury had the highest recurrence alongside upper or lower back injury.
Compared to age-matched, non-contact controls, the prevalence of osteoarthritis was more than twofold greater in former elite rugby code athletes (51% v 22%), and was associated with previous joint injury and surgery. The prevalence of current back pain and severe and regular joint pain was high in all former athletes, particularly former elite rugby code athletes (64% and 80%, respectively).
It is important that efforts are prioritized to reduce the occurrence and improve the longer term monitoring and management of injuries in rugby code athletes at all levels. In addition, strategies are needed to support the specific health needs of players post-retirement.

## Background

Rugby is an intermittent contact team-sport that is played in games of two 40 min halves or two 7 min halves (for rugby sevens), and involves numerous collisions and tackles. There are two codes of rugby that differ according to rules and the number of players on the field; rugby union and rugby league. Rugby union is one of the world’s most popular team contact sports [1, 2], with over 8.5 million athletes playing rugby union across 121 countries [2]. In the United Kingdom (UK) and Ireland alone, more than 2.5 million people play rugby union, over a quarter of whom are teenage athletes, indicating potential growth in future years [2]. Rugby league is played mainly in the United Kingdom (UK), France, Australia, and New Zealand. Despite popularity, the fast-paced, full-contact nature of both codes of rugby results in a higher frequency of injury than in other contact and non-contact sports [1, 3–11].

Rugby code injury rates have been studied prospectively across different countries and levels [3–22]. In elite men’s rugby union, pooled injury incidence has been reported at 81 injuries/1000 player match-hours [3], and at amateur level, 47 injuries/1000 player match-hours [6]. Comparatively, in elite men’s rugby league, pooled injury incidence has been reported at 148 injuries/1000 player match-hours [8] and in amateur rugby league, 62 injuries/1000 player match-hours [4]. Tackling is a common match activity and has repeatedly been found to be the cause of most injuries in rugby union [1, 5, 6, 14, 16, 17] and rugby league [23, 24], and injury risk is also associated with higher cumulative training loads [25]. In response to high-injury rates, efforts have been made to reduce injuries in rugby codes, with programmes such as Tackling Rugby and RugbySmart in New Zealand [26, 27], BokSmart in South Africa [28], and Activate in England [29]. Nonetheless, rates are reported to be three times higher than in American football in terms of injuries per 1000 athlete exposures [12], indicating a greater risk for injury in rugby codes for each individual athlete selected to participate. Rugby code injury rates are also markedly higher than the 27.5 and 2.7 injuries per 1000 player match-hours reported in elite soccer and cricket, respectively [10, 11].

Although exercise is beneficial to overall wellbeing, in some cases, the risks associated with the activity can outweigh the benefits of participation. As a result, it is important to consider the long-term implications of high-injury-rate sports on overall health to ensure that individuals can make informed decisions regarding participation [30]. Despite injury incidence being well-studied in both rugby codes, no study has yet explored total injuries sustained across a rugby code career, and very few have considered the long-term and cumulative effects of injuries on players’ overall physical wellbeing [30, 31]. One study providing a 5-year follow-up on injuries occurring during the 1993–1994 rugby union season found that 35% of players reported temporary or meaningful impacts on their education, employment, family life, or health and general fitness from their rugby injury [31]. Furthermore, a cross-sectional study reported a greater prevalence of osteoporosis (OR 2.69 [95% CI 1.35–5.38]), osteoarthritis (OR 4.00 [95% CI 3.32–4.81]), and joint replacement (OR 6.02 [95% CI 4.66–7.77]) in retired rugby union players than in the general population [30]. Only 45% of participants strongly agreed that, considering the benefits and risks associated, they would recommend elite rugby to their children, relatives or close friends [30].

To date, no study has examined the occurrence of total injuries across entire careers of contact sport athletes, and no study has explored the reported effects on physical wellbeing post-retirement. To facilitate informed choices regarding participation in rugby codes versus other non-contact sports, it is important to fully understand the associated risks in terms of the cumulative injury load (occurrence or recurrence of injuries) and long-term impact of that load across all levels of performance. This study aimed to investigate total injuries, cumulative injury load levels, and reported longer term effects, in retired UK rugby code athletes from both codes compared to retired non-contact sport athletes.

## Methodology

### Study Design and Setting

The current research was a cross-sectional analysis of 254 participants from the UK Rugby Health Project. With a multidisciplinary research focus, the UK Rugby Health Project was initiated in 2016 as an extension to the inaugural New Zealand Rugby Health Project [32] and in response to calls for international efforts to acquire further knowledge and understanding of the health and wellbeing of contact sports athletes. The project was approved by Durham University and Leeds Beckett University Research Ethics Committees, and the study was performed in accordance with the Standards of Ethics outlined in the Declaration of Helsinki. Informed consent was obtained from all individual participants included in the study, and to protect the identity of athletes, this current study was completed anonymously online.

### Study Participants

Former male rugby code athletes and non-contact sport athletes took part in the study and were recruited from September 2016 to December 2018 using past player/athlete associations, printed and televised media reports, word of mouth, and social media. The primary outcome variables for the current study were injury rates. In unpublished data from the New Zealand Rugby Health study, comparisons of injury rates between the two rugby code groups and non-contact sport athletes revealed effect sizes that ranged from 0.2 to 0.6, depending on the site of injury. Using an effect size of 0.4, alpha 0.05, and power 0.8, the estimated sample size for a one tail comparison is 78 per group. Elite rugby code athletes (*n* = 83) had competed at international or national level, and/or at professional or semi-professional level (elite rugby union *n* = 49; elite rugby league *n* = 34). The amateur rugby code group (*n* = 106) played at club or regional level and had not received payment for playing. The majority of the amateur rugby code group were former rugby union players (amateur rugby union *n* = 96; amateur rugby league *n* = 10). The inclusion criteria for the non-contact group were that they may have participated at competitive level (structured and organized sporting events), but must not have taken part in any contact sport post-school. Over half of the retired non-contact athletes reported cricket as their main sport (*n* = 35) and the remainder reported running, swimming or cycling (*n* = 30).

### Procedures

Information on engagement in sport, demographic information, injuries including concussion, and current health and wellbeing were obtained from a general health e-questionnaire. This questionnaire was adapted from the New Zealand Rugby Health study, where it had been developed and administered to obtain meaningful information from 366 former athletes [32]. The adaptations were made to reflect the rugby union and rugby league competition structure in the UK. The questionnaire has six sections (*Section* *1: Demographics*; *Section* *2: Sport Participation*; *Section* *3: Ability to Perform Tasks*; *Section* *4: Injury History*; *Section* *5: Health*, *Lifestyle and Wellbeing*; *Section* *6: Other Details e.g. education*, *relationship status*), a total of 97 questions, and takes approximately 40 min to complete. An injury was defined as a physical problem leading to training and/or match time loss, or which required assessment and/or treatment by a health professional. Reported medical conditions, such as osteoarthritis, were those which had received medical diagnoses. The questionnaire was available online from September 2016 to December 2018. Although the questionnaire could also be accessed as a paper version, this was only utilized by one participant and responses were entered manually into the study database.

### Statistical Analyses

All data were compared between elite rugby codes, amateur rugby codes, and non-contact athletes. We also included sub analyses of data between rugby union and rugby league, and detailed results for each code are provided in the Appendix (Online Appendix 1). The derived variables were categorical (for example, highest performance level) or continuous (for example, age, number of a given injury) (see Data Dictionary, Online Appendix 2). The primary outcome variables were total injuries per athlete, percentage of athletes reporting a given injury, cumulative injury load (see below for definition), surgeries, current physical ailments, and continued impact of an injury. Descriptive data were presented as mean ± standard deviation when normally distributed or median and interquartile range (IQR) where data were skewed. Risk ratios with 95% confidence intervals (CI) for elite and amateur rugby groups were calculated compared to non-contact controls for occurrence of injuries, continued impact of injuries and surgeries. Data were analyzed using SPSS for Windows (IBM SPSS, Version 22, Armonk, New York) and Microsoft Excel (Microsoft Corporation 2016, Version 1902, Redmond, Washington). Statistical significance was *p* < 0.05.

#### Cumulative Injury Load Variable

To control for the right skew and frequent extreme outliers in the number of injuries reported, the injury data separated by individual injury type were presented as ‘cumulative injury load’, as defined in Table 1. The cut points were determined by distinguishing the mean 85th (5 occurrences of one injury) and 95th (10 occurrences of one injury) percentile of total number of injury type reported per rugby code athlete with at least one case of the given injury. This allowed retention of information on recurrent injuries, while creating a more-normalized variable appropriate for statistical analyses. Due to the ordinal nature of the variable, distributions of cumulative injury load were compared using an independent-samples’ Kruskal–Wallis test. When significant differences were found, post hoc pairwise comparisons were performed using a two-tailed Dunn’s test with Bonferroni’s adjustment to control for alpha inflation.

**Table 1:** Cumulative injury load variable

Number of a given injury	Cumulative injury load
0	0
1–4	1
5–9	2
10+	3

#### Surgeries, Current Physical Ailments, and Continued Impact from an Injury

Binomial data were analyzed for the occurrence of surgeries, the presence of ailments, and continued impact from an injury. These data were analyzed using the Chi-square test of independence, or in cases where expected cell counts in the frequency table were less than 5 with the Fisher’s exact test. When significant differences were found, post hoc pairwise Chi-square tests and Fisher’s exact tests with Bonferroni’s Adjustment were performed. Data on the mean number of injuries and surgeries in those with and without joint ailments across all groups were analyzed using an independent-samples *t* test, after using a Q–Q plot to test for approximate normality.

## Results

### Demographic Characteristics

There were no differences in age, retirement age, or years in sport between former rugby union and former rugby league players (Table 2). The injuries most likely to cause retirement were concussion, arm/wrist/hand fractures, disc rupture, or herniation, ACL tear, or back injury in elite rugby (9 to 10%); ACL tear, meniscus tear or knee ligament injury in amateur rugby (8 to 11%), and back injury (7%) in non-contact athletes.

**Table 2:** Participant demographics of elite rugby codes, amateur rugby codes, non-contact, and combined sports participants reported by mean ± standard deviation

	*N*	Age *Range*	Starting age	Retirement age	Average years in the sport
Elite rugby code athletes	83	43.4 ± 9.4 *21.5*–*73.5*	8.8 ± 2.9	33.2 ± 5.6	24.3 ± 6.5
Amateur rugby code athletes	106	48.3 ± 11.0 *24.2*–*82.2*	10.6 ± 3.6	36.4 ± 9.6	25.7 ± 9.3
Non-contact athletes	65	48.7 ± 12.9 *24.3*–*72.9*	10.7 ± 4.8	41.9 ± 12.7	32.0 ± 13.4
Combined	254	46.8 ± 11.3 *21.5*–*82.2*	10.0 ± 3.8	36.3 ± 9.6	26.4 ± 9.7

### Injury Incidence

The median number of injuries per athlete were higher for elite rugby code athletes (39 [IQR 35]) than amateur rugby code athletes (23 [IQR 30], *p* = 0.014) and more than 5 times greater than the non-contact sports group (7.5 [IQR 15], *p* < 0.001). There were no differences in the total number of injuries between former rugby union (median 30 [IQR 30]) and rugby league players (median 28.5 [IQR 39], *p* = 0.712) (see Online Appendix 1 for data). The percentage of participants who experienced a given injury through their main sport are reported in Table 3. Over half of players from both rugby codes had experienced at least one concussion, rib fracture or bruising, arm/hand/wrist fracture, upper or lower back injury, and at least one hamstring or calf strain or tear (Table 3). The only differences between rugby union and rugby league were for elbow injuries (*p* = 0.016) and shoulder injuries (*p* = 0.045) which were more common in rugby league. Statistically significant risk ratios for occurrence of an injury in rugby code athletes compared to non-contact sport athletes, ranged from 1.71 (95% CI 1.11–2.63) to 7.33 (95% CI 1.80–29.91). Risk ratios were highest for concussions (elite rugby code athletes: 3.39 [95% CI 2.08–5.50]); amateur rugby code athletes: 3.16 [95% CI 1.94–5.13]), bicep tears (elite rugby code athletes: 7.33 [95% CI 1.80–29.91]), and MCL injuries (elite rugby code athletes: 4.89 [95% CI 1.54–15.53]).

**Table 3:** Percentage of participants reporting given injury

	Elite rugby codes (ER) (%)	Amateur rugby codes (AR) (%)	Non-contact (NC) (%)	Differences between groups (*p*)
Neck fracture/spinal cord injury	11	2	2	ER v AR *p* = 0.027
Concussion	81	76	24	ER v NC *p* < 0.001; AR v NC *p* < 0.001
Collar bone fracture	19	17	7	ns
Arm/wrist/hand fracture	58	55	33	ER v NC *p* = 0.015; AR v NC *p* = 0.030
Ribs broken/fractured or bruised	64	66	22	ER v NC *p* < 0.001; AR v NC *p* < 0.001
Hip dislocation/fracture	2	3	2	ns
Thigh/leg fracture	12	10	7	ns
Ankle/foot fracture	25	15	17	ns
Shoulder dislocation	43	29	19	ER v NC *p* = 0.009
Elbow dislocation/separation	15	6	6	ns
Knee/patellar dislocation	11	8	9	ns
Biceps/triceps tear	27	13	4	ER v AR *p* = 0.036; ER v NC *p* < 0.001
Hamstring/quad tear	52	40	43	ns
Medial collateral ligament tear	27	15	6	ER v NC *p* = 0.006
Lateral collateral ligament tear	12	7	6	ns
Anterior cruciate ligament tear	21	24	7	AR v NC *p* = 0.030
Posterior cruciate ligament tear	20	3	0	ER v AR *p* < 0.001; ER v NC *p* = 0.003
Meniscus tear	36	28	20	ns
Calf/Achilles tendon tear	33	24	26	ns
Ankle ligament tear	57	46	24	ER v NC *p* < 0.001; AR v NC *p* = 0.021
Hamstring or calf strain or tear	54	51	44	ns
Achilles tendonitis	25	16	24	ns
Disc rupture/herniation	23	7	15	ER v AR *p* = 0.003
Neck burner/numbness	52	29	15	ER v AR *p* = 0.003; ER v NC *p* < 0.001
Neck sprain	41	38	11	ER v NC *p* < 0.001; AR v NC *p* < 0.001
Thigh strain or bruising	60	54	31	ER v NC *p* = 0.003; AR v NC *p* = 0.021
Thumb sprain	60	54	19	ER v NC *p* < 0.001; AR v NC *p* < 0.001
Upper or lower back injury	69	68	54	ns
Eye injury	40	34	19	ER v NC *p* = 0.030
Knee ligament injury	68	48	20	ER v AR *p* = 0.021; ER v NC *p* < 0.001; AR v NC *p* = 0.003

In all cases where significant differences occurred, cumulative injury loads were greater in elite than in amateur rugby code athletes, or non-contact athletes (concussion, arm/hand/wrist fracture, rib injury, shoulder dislocation, biceps/triceps tear, MCL tear, PCL tear, ankle and knee ligament injury, disc rupture or herniation, neck injury, thigh contusion, thumb sprain, and eye injury), and greater in amateur rugby codes than in the non-contact group (concussion, arm/hand/wrist fracture, rib injury, ACL tear, ankle and knee ligament injury, neck injury, thigh contusion, and thumb injury) (Table 4). As with injury impact data, in all cases where significant differences occurred, surgeries were more frequent in elite than in amateur rugby code athletes or non-contact athletes (shoulder dislocation, ACL tear, knee ligament, disc rupture or herniation, and upper/lower back injury), and more frequent in amateur rugby code athletes than in non-contact athletes (ACL injury) (Table 4).

**Table 4:** Cumulative injury load

	Elite rugby codes (ER)	Amateur rugby codes (AR)	Non-contact (NC)	Differences between groups (*p*)
Neck fracture/spinal cord injury	0.11	0.02	0.02	ER v AR *p* = 0.014; ER v NC *p* = 0.048
Concussion	1.27	1.03	0.26	ER v NC *p* < 0.001; AR v NC *p* < 0.001
Collar bone fracture	0.19	0.17	0.07	ns
Arm/wrist/hand fracture	0.70	0.63	0.37	ER v NC *p* = 0.010; AR v NC *p* = 0.040
Ribs broken/fractured or bruised	0.69	0.82	0.24	ER v NC *p* < 0.001; AR v NC *p* < 0.001
Hip dislocation/fracture	0.02	0.03	0.02	ns
Thigh/leg fracture	0.12	0.10	0.07	ns
Ankle/foot fracture	0.26	0.17	0.17	ns
Shoulder dislocation	0.48	0.31	0.20	ER v NC *p* = 0.008
Elbow dislocation/separation	0.15	0.06	0.06	ns
Knee/patellar dislocation	0.11	0.08	0.09	ns
Biceps/triceps tear	0.27	0.13	0.04	ER v AR *p* = 0.019; ER v NC *p* = 0.001
Hamstring/quad tear	0.69	0.51	0.50	ns
Medial collateral ligament tear	0.28	0.15	0.06	ER v NC *p* = 0.003
Lateral collateral ligament tear	0.12	0.07	0.06	ns
Anterior cruciate ligament tear	0.21	0.24	0.07	AR v NC *p* = 0.036
Posterior cruciate ligament tear	0.20	0.03	0.00	ER v AR *p* < 0.001; ER v NC *p* < 0.001
Meniscus tear	0.40	0.29	0.24	ns
Calf/Achilles tendon tear	0.36	0.26	0.30	ns
Ankle ligament tear	0.70	0.58	0.33	ER v NC *p* = 0.002; AR v NC *p* = 0.049
Hamstring or calf strain or tear	0.85	0.71	0.59	ns
Achilles tendonitis	0.33	0.20	0.24	ns
Disc rupture/herniation	0.26	0.07	0.15	ER v AR *p* = 0.003
Neck burner/numbness	0.90	0.39	0.15	ER v AR *p* < 0.001; ER v NC *p* < 0.001
Neck sprain	0.54	0.54	0.15	ER v NC *p* = 0.002; AR v NC *p* = 0.003
Thigh strain or bruising	1.15	0.91	0.48	ER v NC *p* = 0.001; AR v NC *p* = 0.028
Thumb sprain	1.00	0.82	0.22	ER v NC *p* < 0.001; AR v NC *p* < 0.001
Upper or lower back injury	1.28	1.19	0.94	ns
Eye injury	0.48	0.42	0.20	ER v NC *p* = 0.033
Knee ligament injury	0.84	0.56	0.22	ER v AR *p* = 0.014; ER v NC *p* < 0.001; AR v NC *p* = 0.004

### Continued Impact of Previous Injuries

The data on continued impact of injuries are shown in Table 5. Previous upper/lower back injury was attributed to continued impact in all retired athlete groups (Table 5). Significant differences in reported continued impact from a previous injury were observed between the elite rugby code athletes and non-contact athletes for concussion (*p* = 0.003), shoulder dislocation (*p* = 0.003), ACL tear (*p* = 0.003), knee ligament (*p* = 0.009), and neck injury (*p* = 0.012), and between the amateur rugby codes and non-contact groups for concussion (*p* = 0.045), ACL tear (*p* = 0.003), and knee ligament injury (*p* = 0.027). Compared to the non-contact group, the significant risk ratios for continued impact of a previous rugby code-related injury were: shoulder dislocation (elite rugby code athletes: 3.92 [95% CI 1.21–12.74]), knee ligament injury (elite rugby code athletes: 3.80 [95% CI 1.39–10.43]; amateur rugby code athletes: 3.33 [95% CI 1.22–9.10]), meniscus tear (elite rugby code athletes: 2.37 [95% CI 1.10–5.12]), and neck burner or numbness (elite rugby code athletes: 9.68 [95% CI 1.31–71.52]). Former rugby league players were more likely than former rugby union players to report a continued impact for previous arm fracture (*p* = 0.012) and knee ligament injuries (*p* = 0.002). Continued impact from ankle tear was more common in rugby union than in rugby league (*p* = 0.016) (see Online Appendix 1 for additional data).

**Table 5:** Continued impact from a previous injury

	Participants who received surgery for a given injury			Participants still affected by a previous injury		
	Elite rugby codes (%)	Amateur rugby codes (%)	Non-contact (%)	Elite rugby codes (%)	Amateur rugby codes (%)	Non-contact (%)
Neck fracture/spinal cord injury	4	1	0	5	1	0
Concussion	1	5	4	15*	10^#^	0
Collar bone fracture	2	4	2	1	7	0
Arm/wrist/hand fracture	25	15	13	22	16	16
Rib fracture or bruising	2	0	2	2	5	0
Hip dislocation/fracture	2	0	0	2	0	0
Thigh/leg fracture	2	3	0	0	2	0
Ankle/foot fracture	4	2	5	5	3	4
Shoulder dislocation	19*^	7	4	21*	10	5
Elbow dislocation/separation	2	1	4	5	2	2
Knee/patellar dislocation	1	3	2	10	4	2
Biceps/triceps tear	9	3	2	7	2	2
Hamstring/quad tear	1	0	0	9	10	4
MCL tear	9	5	2	9	6	0
LCL tear	2	3	0	2	1	2
ACL tear	17*	13^#^	2	15*	16^#^	0
PCL tear	6	1	0	9	2	0
Meniscus tear	32	18	14	30	15	13
Calf/Achilles tendon tear	7	3	5	12	6	5
Ankle ligament tear	10	2	2	12	13	9
Hamstring or calf strain or tear	2	0	0	6	7	4
Knee ligament injury	20*	14	4	27*	24^#^	7
Achilles tendonitis	2	0	4	5	7	7
Disc rupture/herniation	14^	1	2	12	6	5
Neck burner/numbness	1	0	0	17*	11	2
Neck sprain	4	0	0	14	8	4
Thigh strain or bruising	1	1	0	1	0	2
Thumb sprain	5	3	0	5	10	2
Upper or lower back injury	10^	1	7	38	32	29
Eye injury	7	4	4	6	3	2

*Elite rugby codes > non-contact, ^ elite rugby codes > amateur rugby codes, ^#^amateur rugby codes > non-contact, all *p* < 0.05

### Current Physical Ailments and Surgeries

The proportion of individuals with given ailments was notably high across all groups (Table 6), although former elite rugby code players were more than twice as likely to have received a medical diagnosis of osteoarthritis compared to former non-contact athletes, with a risk ratio of 2.35 (95% CI 1.41–3.91). Reported injuries requiring surgery were greater in former rugby code groups than the non-contact group for shoulder dislocation (elite rugby codes v non-contact, *p* = 0.027), ACL tear (elite rugby codes v non-contact, *p* = 0.012; amateur rugby codes v non-contact, *p* = 0.048), and knee ligament injury (elite rugby codes v non-contact, *p* = 0.018). More surgeries for disc rupture/herniation, shoulder dislocation, and back injury were reported by former elite compared to former amateur rugby code players (*p* = 0.003, *p* = 0.039, *p* = 0.033). By rugby code, more surgeries were reported for back injuries (*p* = 0.035) and knee ligament injuries (*p* = 0.031) in former rugby league compared to rugby union players.

**Table 6:** Percentage of participants currently affected by physical ailments

	Elite rugby code athletes (%)	Amateur rugby code athletes (%)	Non-contact athletes (%)
Back pain	80	75	69
Severe and regular joint pain	64	53	47
Osteoarthritis	51*	36	22

Denotes significance at *p* < 0.05 for *: elite v non-contact

Former athletes reporting medically diagnosed osteoarthritis and/or severe and regular joint pain reported a significantly higher number of total injuries and sport injury-related surgeries (Table 7). The only non-significant difference in the number of total injuries was between those with and without severe and regular joint pain (*p* = 0.149). In all cases, there were more prior injuries and surgeries in those who had osteoarthritis and severe and regular joint pain than in those who did not (Table 7).

**Table 7:** Past sport-related injuries and surgeries in participants (combined rugby and non-contact) with and without joint ailments

	Osteoarthritis		Severe and regular joint pain	
	No	Yes	No	Yes
Joint injuries	3.8	5.5*	3.7	5.0*
Total injuries	27.5	40.2*	28.8	35.3
Total surgeries	1.4	4.5*	1.4	3.5*

* denotes significance at *p* < 0.05 between those with and without joint ailment

## Discussion

The significant findings of this study were first, that across multiple injury types, past participation in rugby codes, particularly at elite level, is associated with a higher number of injuries, recurrent injuries, a continued impact of previous injuries post-retirement, and more than a twofold greater risk for osteoarthritis. Second, that concussion injury was the most commonly reported injury in both elite and amateur rugby codes, and was the injury with the highest cumulative load, indicating the highest rate of recurrence. Third, that the prevalence of current back pain and severe and regular joint pain was high for all former athletes, particularly former elite rugby code players. These data provide a strong basis for future research and intervention, for informing on player welfare both during, and post career, and offers important information to national governing bodies, and athletes themselves, on the cumulative injury load and risks in rugby codes.

Our findings add to the knowledge base on lifetime injuries sustained by rugby code athletes and suggest that for each season played, individuals are at risk of sustaining at least one injury. There were a high number of reported injuries per player, equivalent to 1.6 injuries/season in former elite rugby code players and 0.9 injuries/season in former amateur rugby code players compared with 0.2 injuries/season in former non-contact athletes. Our findings support those from rugby union injury surveillance studies reporting an average of 1.8 match injuries/player/season in the English Premiership [19], and from English Super League, reporting an average of 41 injuries/club/season [7]. It should also be considered that injury risk exposure levels will be greater for rugby code players at elite, than at amateur level, which is likely to explain the disparity in injuries/player by performance level.

The most common injury reported was concussion, followed by injuries to the back and to the knee ligament. The most frequently reported injury (total number of a given injury) was also concussion, followed by thumb sprain, and thigh contusion. This reflects recent injury surveillance data [7, 20, 21], and suggests that approximately 80% of rugby code players will experience at least one concussion at some point during their playing career, which, in the current study, spanned an average of 24–25 years. This is notably high in comparison to the data from earlier (pre-2005) injury surveillance studies [19, 22]. In the current study, past players were on average, exposed to rugby union or rugby league prior to 2010 when reported concussions in both codes were lower than for other injuries. The introduction of concussion laws (2012 for rugby union and 2014 for rugby league) is likely to have improved the reporting of concussion and there has been an improvement in the awareness of concussion as an injury. It is plausible to explain our findings based on increased awareness of concussion amongst past players retrospectively reflecting on their own experience of this injury in the context of current definitions and improved awareness. In addition, there is growing concern about the effects of cumulative concussive injuries to the brain [33, 34], and in support of this concern, our findings indicate that concussion is the most common injury with highest recurrence across a rugby code career.

Although it has been suggested that increased skills and technical proficiency can reduce the risk of certain types of injuries [35], we observed that in all cases where differences were statistically significant, injury numbers were higher in elite than in amateur rugby code players. We also found a higher recurrence of numerous injuries in elite rugby codes compared with amateur rugby codes (neck injury, biceps or triceps tear, knee ligament injury, PCL tear, and disc rupture or disc herniation). This suggests that there is no injury type for which there is a protective effect as skill level increases. Instead, a greater intensity of play, a more frequent exposure to risk of injury, and the financial need or desire to return to high-level sport, are likely to be more plausible explanations. The monitoring of injuries at the player level rather than at club level could be one approach to improving the management of injury for individualized player welfare. This could include a system by which the individual player’s injury history can be followed as they move across professional club contracts, and specific strength and conditioning and sports therapy strategies prescribed to help prevent recurrence of injury. In addition, the prescribing of individual player load and recovery relative to how the player is feeling or performing on a particular week would represent good practice.

Surgery data provided information on the impact and severity of the injuries experienced. The most common surgery reported was for meniscus tears, with approximately one-third of elite rugby athletes having at least one meniscus surgery and more likely to report long-term impact from this injury (RR 2.370, 95% CI 1.098–5.119). Although at a lower rate than in elite rugby code athletes, meniscus surgeries were also among the most common surgeries for amateur rugby code (18%) and non-contact (14%) athletes. This may reflect the involvement of the knee in sports that involve twisting and turning, including cricket, which was the main sport of our non-contact group. Overall, our findings do indicate that elite rugby code athletes are at a greater risk for sport-related surgeries than amateur rugby code and non-contact athletes, with surgeries more frequently involving the knee, shoulder and back (knee ligament injuries, ACL tears, shoulder dislocations, and disc rupture/herniation). Although cumulative injury loads were higher in amateur rugby code than non-contact athletes for several types of injury, the only significant difference in the occurrence of surgeries between these two groups was for ACL tears. This finding may indicate that there are notably more injuries in amateur rugby code athletes than non-contact athletes, but these injuries may not be to the extent that require surgery. However, there are numerous factors impacting upon whether or not an athlete has surgery for a given injury, with some injury types, such as ACL tears, more likely to require surgical treatment than others, and other factors, including the need or desire to return to high-level sport which is greater in professional athletes.

To understand the overall impact of injuries and surgeries, it is important to consider not only the injuries that occurred but also the long-term impact of those injuries, as evidenced by current ailments and whether injuries are still impacting these athlete’s, post-retirement. More retired elite rugby code players than non-contact athletes reported that they were still regularly affected by past sport-related injuries. Specifically, retired elite rugby code players attributed previous concussion, arm/hand/wrist fractures, neck burners/numbness injuries, shoulder dislocations, ACL tears, and knee ligament injuries, to an adverse impact on their current health status. Retired amateur rugby code players were also more likely to be still affected by concussions and general knee ligament injuries, specifically ACL tears, suggesting that the elevated risk of long-term injury impact exists at all levels of the sport. It should be considered that study participants reported injury severity in terms of surgeries and long-term impact, rather than recovery time, and therefore, it is not possible to compare these data to other published reports of injury severity in terms of exact time loss.

According to the Global Burden of Disease report, chronic back pain and osteoarthritis are leading causes of disability worldwide [36]. The occurrence of back pain and severe and regular joint pain was prominent amongst all former athlete groups, and the greatest occurrence was in elite rugby code athletes. It is unclear if the high prevalence of back pain in former rugby code players is related to vertebral injuries during their playing career [37]. However, it is notable that the reported prevalence of back pain (69–80%) or severe and regular joint pain (47–64%) in former athletes is higher than the prevalence of chronic pain or low back pain in the UK general population (43–48%) and specific to males (31.0–48.9%) [38, 39]. There is a need for future research to examine the underlying causes and implications of chronic pain in former athletes and to develop effective strategies for pain management in this population.

In addition to pain, the prevalence of medically diagnosed osteoarthritis was 2.3-fold higher in former elite rugby code than former non-contact athletes. Davies et al. [30] recently reported a fourfold higher prevalence of osteoarthritis in retired elite rugby union players compared to a general population cohort [30]. Although these results do not infer causation, reported medically diagnosed osteoarthritis was associated with previous joint injury, the total number of injuries, and previous sport injury-related surgery. These findings confirm that prior injury is a precursor to osteoarthritis [40], and together, these data suggest that retired rugby code athletes have an increased risk of developing osteoarthritis, which, at least in part, may be due to their higher rates of injury. As such, it is plausible to suggest that reducing the total number of sport-related injuries and surgeries in rugby union and rugby league may reduce the risk for osteoarthritis in later life.

This was the first study to describe the cumulative injury history and current physical health in former contact and non-contact sport athletes. There are several considerations to note hen interpreting our findings. First, as with similar studies involving the recruitment of volunteers, the study was subject to non-response or selection bias, which may mean that those most affected by injuries responded to the invitation to participate or that those affected by particularly serious injuries were unable to participate and, therefore, results may not be entirely generalizable to all rugby players. The inclusion of both elite and amateur level former rugby code athletes lends to increase the applicability of the study findings. Second, we acknowledge that the sample sizes may have been underpowered to detect some differences in reported injuries between groups for example, when comparing surgeries. Third, given the inclusion criteria for the study, a range of participant ages and sport exposure was expected. On average, participants had over 25 years in their sport, which suggests a large exposure for most participants. Injuries sustained during school sport were not within the scope of the current study. It is possible that former athletes in the non-contact sport group had taken part in rugby codes or other contact sports during their school years. Likewise, injuries sustained during other sports outside of rugby codes or the main sport of the non-contact athletes were not included in the study. Fourth, as with all studies involving an element of information recall, there is potential for recall bias, and so, there is a risk for underestimation or overestimation of injury occurrence. We took strategies to mitigate this risk by providing clear definitions of injuries, including concussion, and current conditions, such as medically diagnosed osteoarthritis. Finally, it was not possible to examine injury recovery time and time lost, and so, data cannot be directly compared with published reports of injury severity in terms of player-days absence and exact risk exposure. Nonetheless, our findings are consistent with prospective injury surveillance studies which report higher injury rates per 1000 player match-hours in rugby than in non-collision-based sports [1, 9].

## Conclusion

In conclusion, our findings indicate that elite rugby code athletes experience notably more injuries and surgeries over the course of their playing careers than non-contact athletes. In addition, elite rugby code athletes appeared to be at an elevated risk for injuries that continued to affect them post-retirement. Although to a lesser extent than elite level rugby codes, amateur rugby code athletes also experienced more injuries and surgeries, and were more likely to have long-term effects of knee ligament injuries than non-contact athletes. Given the high number of reported concussions and their recurrence, and the association between current musculoskeletal conditions and previous injuries during a player’s career, governing body efforts should continue to be prioritized to reduce the occurrence of rugby union and rugby league injuries in both the elite and amateur game. Importantly, strategies should also be developed for supporting the specific physical health needs of rugby code athletes’ post-retirement.

## Electronic Supplementary Material

Below is the link to the electronic supplementary material.

Supplementary material 1 (DOCX 27 kb)

Supplementary material 2 (XLSX 19 kb)
