# Supplementary material for: Cumulative Sport-Related Injuries and Longer Term Impact in Retired Male Elite- and Amateur-Level Rugby Code Athletes and Non-contact Athletes: A Retrospective Study
Source: Sports Med. 2020 Jul 16;50(11):2051–61. doi: 10.1007/s40279-020-01310-y (PMC7575474; doi:10.1007/s40279-020-01310-y)
Supplement: Supplementary file 1 — Supplementary material 1 (DOCX 27 kb) [file 40279_2020_1310_MOESM1_ESM.docx]

**Appendix**

Rugby League & Rugby Union Tables

*Table 1. Participant demographics of rugby league and rugby union reported by mean with ± standard deviation.*

|  | **N** | **Age** | **Starting age** | **Retirement age** | **Average years in the sport** | **Percent Elite** |
| --- | --- | --- | --- | --- | --- | --- |
| Rugby League | 44 | 46.9 ± 10.1 | 10.0 ± 3.6 | 33.9 ± 6.2 | 24.0 ± 7.2 | 77.3% |
| Rugby Union | 145 | 45.9 ± 10.8 | 9.8 ± 3.4 | 35.3 ± 8.7 | 25.5 ± 8.6 | 33.8% |

*Table 2. Percentage of participants reporting given injury*

|  | **Rugby Union** | **Rugby League** | ***Difference* *(p)*** |  |
| --- | --- | --- | --- | --- |
| Neck fracture/Spinal cord injury | 5% | 9% | ns |  |
| Concussion | 79% | 77% | ns |  |
| Collar bone fracture | 19% | 14% | ns |  |
| Arm/Wrist/Hand fracture | 55% | 61% | ns |  |
| Ribs broken/fractured or bruised | 67% | 61% | ns |  |
| Hip dislocation/fracture | 4% | 0% | ns |  |
| Thigh/Leg fracture | 11% | 9% | ns |  |
| Ankle/foot fracture | 17% | 27% | ns |  |
| Shoulder dislocation | 31% | 48% | p=0.045 |  |
| Elbow dislocation/separation | 6% | 20% | p=0.016 |  |
| Knee/Patellar dislocation | 9% | 9% | ns |  |
| Biceps/Triceps tear | 18% | 20% | ns |  |
| Hamstring/Quad tear | 49% | 34% | ns |  |
| Medial collateral ligament tear | 22% | 16% | ns |  |
| Lateral collateral ligament tear | 11% | 2% | ns |  |
| Anterior cruciate ligament tear | 20% | 32% | ns |  |
| Posterior cruciate ligament tear | 11% | 9% | ns |  |
| Meniscus tear | 34% | 23% | ns |  |
| Calf/Achilles tendon tear | 28% | 27% | ns |  |
| Ankle ligament tear | 52% | 48% | ns |  |
| Hamstring or calf strain or tear | 53% | 50% | ns |  |
| Achilles Tendonitis | 22% | 14% | ns |  |
| Disc rupture/Herniation | 13% | 16% | ns |  |
| Neck burner/numbness | 41% | 32% | ns |  |
| Neck sprain | 42% | 30% | ns |  |
| Thigh strain or bruising | 57% | 57% | ns |  |
| Thumb sprain | 56% | 59% | ns |  |
| Upper or Lower back injury | 67% | 73% | ns |  |
| Eye Injury | 33% | 45% | ns |  |
| Knee ligament injury | | 54% | 66% | ns |

|  | **Rugby Union** | **Rugby League** | **Difference (p)** |  |
| --- | --- | --- | --- | --- |
| Neck fracture/Spinal cord injury | 0.05 | 0.09 | ns |  |
| Concussion | 1.11 | 1.23 | ns |  |
| Collar bone fracture | 0.19 | 0.14 | ns |  |
| Arm/Wrist/Hand fracture | 0.63 | 0.75 | ns |  |
| Ribs broken/fractured or bruised | 0.80 | 0.64 | ns |  |
| Hip dislocation/fracture | 0.04 | 0 | ns |  |
| Thigh/Leg fracture | 0.11 | 0.09 | ns |  |
| Ankle/foot fracture | 0.18 | 0.30 | ns |  |
| Shoulder dislocation | 0.34 | 0.52 | p=0.046 |  |
| Elbow dislocation/separation | 0.06 | 0.20 | p=0.006 |  |
| Knee/Patellar dislocation | 0.09 | 0.09 | ns |  |
| Biceps/Triceps tear | 0.18 | 0.20 | ns |  |
| Hamstring/Quad tear | 0.64 | 0.43 | ns |  |
| Medial collateral ligament tear | 0.23 | 0.16 | ns |  |
| Lateral collateral ligament tear | 0.11 | 0.02 | ns |  |
| Anterior cruciate ligament tear | 0.20 | 0.32 | ns |  |
| Posterior cruciate ligament tear | 0.11 | 0.09 | ns |  |
| Meniscus tear | 0.36 | 0.25 | ns |  |
| Calf/Achilles tendon tear | 0.31 | 0.27 | ns |  |
| Ankle ligament tear | 0.65 | 0.57 | ns |  |
| Hamstring or calf strain or tear | 0.82 | 0.61 | ns |  |
| Achilles Tendonitis | 0.29 | 0.16 | ns |  |
| Disc rupture/Herniation | 0.15 | 0.16 | ns |  |
| Neck burner/numbness | 0.64 | 0.55 | ns |  |
| Neck sprain | 0.61 | 0.32 | ns |  |
| Thigh strain or bruising | 1.01 | 1.05 | ns |  |
| Thumb sprain | 0.88 | 0.95 | ns |  |
| Upper or Lower back injury | 1.23 | 1.23 | ns |  |
| Eye Injury | 0.40 | 0.61 | ns |  |
| Knee ligament injury | | 0.63 | 0.84 | ns |

*Table 3. Cumulative injury load*

*Table 4. Continued impact from a previous injury*

|  | *Participants who received surgery for a given injury* | | | *Participants still affected by a previous injury* | | |
| --- | --- | --- | --- | --- | --- | --- |
|  | **Rugby Union** | **Rugby League** | ***Difference (p)*** | **Rugby Union** | **Rugby League** | ***Difference (p)*** |
| Neck fracture/Spinal cord injury | 2% | 2% | ns | 1% | 7% | ns |
| Concussion | 4% | 2% | ns | 12% | 11% | ns |
| Collar bone fracture | 3% | 5% | ns | 6% | 0% | ns |
| Arm/Wrist/Hand fracture | 19% | 20% | ns | 15% | 32% | p=0.012 |
| Rib fracture or bruising | 0% | 5% | ns | 4% | 5% | ns |
| Hip dislocation/fracture | 1% | 0% | ns | 1% | 0% | ns |
| Thigh/Leg fracture | 3% | 2% | ns | 1% | 0% | ns |
| Ankle/foot fracture | 3% | 2% | ns | 4% | 2% | ns |
| Shoulder dislocation | 10% | 18% | ns | 13% | 20% | ns |
| Elbow dislocation/separation | 2% | 0% | ns | 2% | 7% | ns |
| Knee/Patellar dislocation | 2% | 2% | ns | 6% | 9% | ns |
| Biceps/Triceps tear | 7% | 0% | ns | 4% | 5% | ns |
| Hamstring/Quad tear | 1% | 0% | ns | 11% | 5% | ns |
| MCL tear | 6% | 9% | ns | 8% | 2% | ns |
| LCL tear | 2% | 5% | ns | 2% | 0% | ns |
| ACL tear | 13% | 23% | ns | 14% | 20% | ns |
| PCL tear | 3% | 5% | ns | 5% | 5% | ns |
| Meniscus tear | 25% | 23% | ns | 23% | 16% | ns |
| Calf/Achilles tendon tear | 4% | 7% | ns | 11% | 2% | ns |
| Ankle ligament tear | 5% | 7% | ns | 16% | 2% | p=0.016 |
| Hamstring or calf strain or tear | 1% | 0% | ns | 6% | 7% | ns |
| Knee ligament injury | 13% | 27% | p=0.031 | 21% | 29% | p=0.020 |
| Achilles tendonitis | 1% | 2% | ns | 8% | 0% | ns |
| Disc rupture/Herniation | 6% | 9% | ns | 9% | 7% | ns |
| Neck burner/numbness | 0% | 2% | ns | 15% | 9% | ns |
| Neck sprain | 1% | 5% | ns | 11% | 9% | ns |
| Thigh strain or bruising | 1% | 2% | ns | 1% | 0% | ns |
| Thumb sprain | 4% | 2% | ns | 7% | 9% | ns |
| Upper or lower back injury | 3% | 11% | p=0.035 | 34% | 39% | ns |
| Eye Injury | 4% | 9% | ns | 4% | 5% | ns |

*Table 5. Percentage of participants currently affected by physical ailments*

|  | **Rugby Union** | **Rugby League** | ***Difference (p)*** |
| --- | --- | --- | --- |
| Back pain | 75% | 82% | ns |
| Severe and regular joint pain | 57% | 61% | ns |
| Osteoarthritis | 37% | 59% | p=0.010 |
